# Analysis of global stock market development—Integration of clustering, classification, and shapley values

**DOI:** 10.1371/journal.pone.0326809

**Published:** 2025-06-24

**Authors:** Marcin Stawarz

**Affiliations:** Department of Applied Informatics and Mathematics in Economics, Faculty of Economic Sciences and Management, Nicolaus Copernicus University in Torun, Torun, Poland; Central Bank of Brazil, BRAZIL

## Abstract

This study aims to analyze the development of global stock exchanges by integrating clustering, classification, and Shapley Values to identify growth patterns and understand the differences in market characteristics and dynamics. The research applies the K-means algorithm for clustering, which enables the segmentation of exchanges based on their similarities. This is followed by using the random forest algorithm to classify these clusters and evaluate the importance of various features. Shapley Values are employed to interpret the contribution of individual variables to the model’s predictions, considering all possible combinations of features. The empirical analysis is based on data from 82 stock exchanges worldwide, sourced from organizations such as the World Federation of Exchanges and the International Monetary Fund. Key variables used include market capitalization, trading value, the number of listed companies, and share turnover velocity. The results highlight the significant heterogeneity among exchanges, with major markets like those in China and the United States forming distinct clusters due to their size, capitalization, and high trading activity. This distinction underscores their dominant position in the global financial landscape. Moreover, exchanges that have emerged from mergers, such as Euronext and NASDAQ Nordic, demonstrate superior characteristics compared to their peers, indicating that consolidation can be an effective strategy for competing with larger markets and enhancing global competitiveness. The study’s findings show that integrating clustering, classification, and Shapley Values is a robust approach for uncovering complex structures within financial markets. This approach provides deeper insights for market participants and policymakers into the growth patterns and strategic positioning of stock exchanges, offering valuable implications for future market development and competition strategies.

## Introduction

Stock exchanges are fundamental components of the global financial system, providing platforms for companies to raise capital and for investors to trade securities [1]. They facilitate the allocation of resources, promote economic growth, and contribute to financial stability by enabling price discovery and liquidity provision [2,3]. The performance and development of stock exchanges have significant implications for the broader economy, influencing investment decisions, corporate financing, and wealth distribution [4].

In recent years, the landscape of global stock exchanges has experienced profound changes driven by globalization, technological advancements, and regulatory reforms [5]. The integration of financial markets has intensified competition among exchanges, prompting them to innovate and adapt to evolving market dynamics [6]. Technological innovations such as electronic trading platforms, high-frequency trading, and blockchain technology have transformed trading practices, enhancing efficiency but also introducing new challenges related to market stability and fairness [7,8].

Understanding the development patterns and competitive dynamics of stock exchanges is crucial for policymakers, regulators, and market participants [9]. Identifying the factors that contribute to the success or underperformance of exchanges can inform strategies to enhance market efficiency, liquidity, and resilience [10]. Key aspects influencing stock exchange development include market liquidity, trading volume, technological infrastructure, regulatory environment, and the degree of market integration [11,12]. A significant trend in the evolution of stock exchanges is the consolidation through mergers and acquisitions [13]. Exchanges such as Euronext and Nasdaq Nordic and Baltic have emerged from the amalgamation of multiple national exchanges, aiming to achieve economies of scale, expand their global reach, and enhance competitiveness [14,15]. Consolidation can lead to increased efficiency and liquidity but may also raise concerns about market concentration and systemic risks [16]. The adoption of advanced technologies and trading mechanisms has also played a critical role in shaping stock exchange development [17]. The implementation of electronic trading systems and algorithmic trading has improved market efficiency and accessibility but has introduced complexities related to market microstructure and potential systemic vulnerabilities [18,19]. Regulatory bodies face the challenge of overseeing these rapidly evolving markets to ensure transparency, fairness, and stability [20]. The recent COVID-19 pandemic has underscored the importance of understanding stock market dynamics, as it led to unprecedented volatility and challenges in financial markets globally [21]. Studies have shown that the pandemic affected different sectors to varying degrees, emphasizing the need for sectoral analysis in assessing stock market stability [22]. For instance, Buszko et al. [23] conducted a sectoral approach to evaluate the pandemic’s impact on stock market stability, revealing varied effects across different industries.

Several studies have explored the determinants of stock exchange development and competitiveness. Pagano [24] analyzed the factors influencing financial market growth, highlighting the role of legal and regulatory frameworks. La Porta et al. [25] emphasized the importance of investor protection laws and corporate governance standards in fostering market development. Levine and Zervos [26] investigated the relationship between stock market development and economic growth, finding a positive correlation between the two. Technological innovation is a critical driver of stock exchange efficiency. Hasan et al. [27] examined the impact of automation on stock exchange performance, finding that technological advancements enhance trading efficiency and liquidity. Hendershott et al. [28] studied the effects of algorithmic trading on market quality, concluding that it improves liquidity but may also increase volatility under certain conditions. The consolidation of stock exchanges has been extensively researched. Nielsson [29] studied the merger of European exchanges and its impact on market liquidity, finding that consolidation generally enhances liquidity. Orzeszko and Stawarz [15] utilized cluster analysis to examine the development of European stock markets, highlighting disparities and similarities among them. Their findings suggest that clustering methods can effectively identify patterns and group exchanges based on performance indicators. Clustering and classification methods are valuable tools in financial research for identifying patterns and grouping entities based on similarities. Jain [30] provided an overview of data clustering techniques and their applications. In the context of stock markets, clustering has been used to segment markets based on risk profiles [31], trading behaviors [32], and financial performance [33]. Machine learning techniques, such as random forests introduced by Breiman [34], have been utilized for tasks like credit scoring [35], bankruptcy prediction [36], and stock price prediction [37], owing to their ability to model complex interactions. Interpretability of machine learning models is crucial in finance, where decision-making requires transparency and understanding of model outputs [38]. Shapley values, derived from cooperative game theory, have been employed to attribute the contribution of each feature to the model’s predictions [39]. Lundberg and Lee [40] developed SHAP (SHapley Additive exPlanations), a unified approach using Shapley values for interpreting complex models, enhancing the ability to explain predictions in a consistent manner. Recent studies have contributed to understanding financial market dynamics using advanced analytical methods. For example, Orzeszko and Piotrowski [41] applied tree-based algorithms to predict robo-advisory acceptance in banking services, demonstrating the effectiveness of machine learning methods in financial contexts. Another study by Buszko et al. [23] assessed the stability of stock markets during the COVID-19 pandemic using clustering techniques, emphasizing the importance of sectoral analysis.

Despite advancements in understanding stock exchange dynamics, several controversies persist. The impact of consolidation on market efficiency and competition remains debated. While some studies advocate that consolidation leads to enhanced liquidity and reduced transaction costs [29], others argue that it may result in monopolistic structures, reducing competition and potentially harming market participants [42]. Concerns about systemic risks associated with large, interconnected exchanges have also been raised [43]. The adoption of high-frequency and algorithmic trading presents another area of contention. While these technologies have improved market efficiency, they have been associated with flash crashes and increased market volatility [44]. The “Flash Crash” of May 6, 2010, highlighted the potential instability of electronic markets and the challenges in regulatory oversight [45]. Balancing innovation with the potential systemic risks introduced by technological advancements remains a critical concern [46]. The use of complex machine learning models in financial analysis introduces challenges related to interpretability and transparency [47]. Regulators and practitioners are concerned about the “black-box” nature of some models, which can obscure the reasoning behind predictions and hinder accountability [48–50]. This has led to an emphasis on developing interpretable models and methods to explain model outputs [40,51].

Recent work has already applied SHAP explanations to market-level problems. Babaei et al. used SHAP to optimise crypto-asset portfolios and showed that feature attributions improve allocation transparency [41]. Giudici, Polinesi & Spelta built network models for robo-advisory portfolios and interpreted edge centralities with SHAP values [42]. More recently, Ahelegbey & Giudici introduced NetVIX, a network-based volatility index whose drivers are likewise revealed through SHAP analysis [43]. All three studies operate at the individual asset or network-edge level and employ supervised or community-detection approaches. By contrast, the present paper (i) clusters entire stock exchanges using unsupervised methods, (ii) constructs a classifier that generalises those clusters, and (iii) explains group membership with SHAP—thereby extending explainable clustering from securities to the market level and uncovering structural commonalities among exchanges worldwide.

The overarching aim of this study is to analyze the development patterns of global stock exchanges by integrating clustering methods, classification algorithms, and interpretability techniques. Specifically, the objectives are to:

- Identify distinct clusters of stock exchanges based on key financial indicators, revealing underlying structures and similarities among exchanges worldwide.
- Develop a robust classification model using random forests that can accurately assign exchanges to the identified clusters, facilitating predictive analysis and assessment of new or emerging exchanges.
- Interpret the classification results using Shapley values, providing insights into the significance of individual features and their impact on the classification outcomes, thereby enhancing the transparency and applicability of the model.

By achieving these objectives, the study contributes to the existing literature by offering a comprehensive methodology that not only classifies stock exchanges but also elucidates the factors driving their development and competitiveness. This integrative approach addresses the need for both analytical rigor and interpretability in financial modeling.

## Materials and methods

A comprehensive methodological framework was employed, integrating data collection, preprocessing, variable selection, clustering using the k-means algorithm, classification with a random forest model, and interpretation of results through Shapley values. This approach facilitated an in-depth analysis of global stock exchanges, enabling the identification of development patterns and key factors influencing their positions within the global financial system.

The dataset comprised 82 stock exchanges from various regions worldwide, representing a diverse range of economic development levels. Data pertaining to the year 2023 were utilized to ensure the most recent and relevant financial information. Authoritative sources were consulted to ensure accuracy. The World Federation of Exchanges (WFE) provided detailed statistics on stock exchanges, including market capitalization, trading volumes, number of listed companies, and other essential financial indicators [52]. The International Monetary Fund (IMF) supplied macroeconomic data such as Gross Domestic Product (GDP) and population figures, enabling the calculation of relative financial metrics [53]. Additional data were obtained directly from the official websites and annual reports of the respective stock exchanges to supplement and verify the collected information. The United Nations Population Division provided updated population data for calculating per capita indicators [54].

An initial set of 13 variables was selected based on their relevance to stock exchange performance and data availability. These variables included market capitalization, market capitalization to GDP ratio, value traded (electronic order book total), value traded to GDP ratio, share turnover velocity, capitalization per listed company, number of listed companies (total), foreign listings ratio, domestic listings per capita, number of domestic listed companies, number of foreign listed companies, number of new listings through IPO, and number of trades (EOB). These variables capture various aspects of stock exchange performance, including size, liquidity, trading activity, internationalization, and market dynamism [55–57].

To ensure comparability across variables with different scales and units, all numerical variables were standardized using z-scores:


$$z_{i}=\frac{x_{i}-\mu }{\sigma },$$ (1)

where $z_{i}$ is the standardized value, $x_{i}$ is the original value, μ is the mean of the variable, and σ is the standard deviation [58]. This standardization centers the data at zero and scales it to have unit variance, which is essential for distance-based clustering methods like k-means [59].

Outliers can significantly affect clustering results; therefore, the Mahalanobis distance was used to detect multivariate outliers [60]. The Mahalanobis distance $D^{2}$ is calculated as:


$$D^{2}=(x-\mu {)}^{⊤}S^{-1}(x-\mu )$$ (2)

where x is the observation vector, μ is the mean vector, and S is the covariance matrix. Observations with $D^{2}$ exceeding the critical value from the chi-square distribution at a significance level of 0.001 were considered potential outliers and were examined further [61].

To enhance clustering performance and interpretability, variable selection was performed with the objective of maximizing the average silhouette score [62]. The initial set of 13 variables was iteratively tested, and variables were selected based on their contribution to cluster separation. After evaluation using the silhouette score, seven variables were selected: market capitalization, market capitalization to GDP ratio, value traded (EOB total), value traded to GDP ratio, share turnover velocity, capitalization per listed company, and number of trades (EOB). These variables provided the highest silhouette score, indicating a better-defined clustering structure [63].

The k-means clustering algorithm was employed to identify natural groupings among stock exchanges based on the selected financial indicators [30]. This algorithm partitions n observations into k clusters, where each observation belongs to the cluster with the nearest mean, serving as the cluster’s centroid [64]. The algorithm seeks to minimize the within-cluster sum of squares (WCSS), defined as:


$$\begin{array}{c} J=\sum_{i=1}^{k}\sum_{x_{j}\in C_{i}}\;\|x_{j}\;-{\;\mu_{i}\|}^{2}, \end{array}$$ (3)

where J  is the total within-cluster variance, $C_{i}$ is the set of observations in cluster i , $x_{j}$ is the j-th observation in cluster i, and $\mu_{i}$ is the centroid of cluster i [65].

The algorithm involves initializing centroids, assigning each observation to the nearest centroid based on the Euclidean distance, recalculating centroids as the mean of all observations assigned to each cluster, and repeating the process until assignments no longer change or the change in the objective function J is below a predefined threshold [66]. While k-means is advantageous due to its simplicity and computational efficiency, it is sensitive to initial centroid positions and requires specifying the number of clusters k in advance [67].

Selecting an appropriate number of clusters is crucial for meaningful clustering results. Two methods were employed: the elbow method and the silhouette score. The elbow method involves plotting the WCSS against a range of k values and identifying the point where the rate of decrease sharply changes, suggesting an optimal k where adding more clusters does not significantly reduce the WCSS [68]. The silhouette score measures how similar an observation is to its own cluster compared to other clusters, defined as:


$$s(i)=\frac{b(i)-a(i)}{\max\text{\textbackslash{}\{}a(i),b(i)\text{\textbackslash{}}}\}$$ (4)

where a(i) is the average distance between i and all other points in the same cluster, and b(i) is the minimum average distance from i to points in a different cluster [69].

A random forest classifier was used to model the relationship between the stock exchange features and the cluster labels obtained from the k means clustering [34]. Random forest is an ensemble learning method that constructs multiple decision trees and aggregates their predictions, introducing randomness through bootstrap aggregation and feature randomness [70]. The algorithm involves generating bootstrap samples, constructing decision trees using random subsets of features, and aggregating predictions by taking the mode of the predictions from all trees [71]. In this study, the classifier’s primary goal was to facilitate the computation of Shapley values for interpreting feature importance, rather than for predictive purposes [72].

Shapley values, derived from cooperative game theory, were employed to quantify the contribution of each feature to the prediction of the model [39]. For a model f and an instance x, the Shapley value $\phi_{i}$ for feature i is calculated as:


$$\begin{array}{c} \phi_{i}=\sum_{S\subseteq N\setminus \text{\textbackslash{}\{}i\text{\textbackslash{}}}\}\frac{|S|!\;\left(|N|-|S|-1\right)!}{|N|!}\left[f\left(S\cup \text{\textbackslash{}\{}i\text{\textbackslash{}}\}\right)-f(S)\right], \end{array}$$ (5)

where N is the set of all features, S is a subset of features not containing i, and n is the total number of features [73]. This formula calculates the average marginal contribution of feature i across all possible subsets. Since the exact computation of Shapley values is computationally intensive ($O(2^{n}\backslash )$complexity), the Tree SHAP algorithm from the SHAP (SHapley Additive exPlanations) library was employed, which efficiently computes exact Shapley values for tree-based models like random forests [40].

The key steps involved training the random forest classifier using the selected features and cluster labels, computing Shapley values for each feature and instance, and performing both global interpretation (aggregating Shapley values across all instances to determine overall feature importance) and local interpretation (analyzing Shapley values for individual instances to understand specific model decisions).

The methodological framework integrated variable selection, clustering, classification, and interpretability techniques to provide a comprehensive analysis. By optimizing the set of variables to maximize the silhouette score, the clustering captured meaningful patterns in the data. The k means clustering identified natural groupings among stock exchanges based on selected financial indicators. The random forest classifier modeled the relationship between features and cluster labels, facilitating interpretation using Shapley values. The interpretability of the model through Shapley values provided insights into feature importance and contributions, enhancing model transparency and understanding.

This integrative approach allowed not only the identification of distinct clusters but also an understanding of the underlying factors influencing stock exchange development and competitiveness. By focusing on the interpretability of the model, valuable insights were gained into how specific financial indicators contribute to the positioning of exchanges within the global financial landscape.

The study was carried out entirely in Python 3.11 using four open‑source libraries that together cover machine‑learning, visual diagnostics, statistical routines and interpretability: scikit‑learn [74], Yellowbrick [75], SciPy [76] and SHAP [40]. Every raw data file (CSV) and an executable Jupyter notebook (research_code.ipynb) capable of regenerating all tables and figures have been archived under a CC0 waiver at https://doi.org/10.18150/OELMLK, guaranteeing unrestricted reuse and full reproducibility. Fig 8 was also generated entirely by the authors using Geopandas and public domain data from Natural Earth (http://www.naturalearthdata.com), and is free of any copyright restrictions.

Workflow at a glance:

1. Data acquisition – World Federation of Exchanges (market statistics), International Monetary Fund (GDP, population), official exchange reports (supplementary indicators) and United Nations Population Division (demographics).
2. Variable definition – compile 13 candidate financial indicators describing size, liquidity and dynamism of each exchange.
3. Pre‑processing – impute sporadic gaps and standardise all variables with z‑scores.
4. Outlier screening – flag extreme observations via Mahalanobis distance.
5. Feature selection – forward search maximising the mean silhouette score; retain the 7 most discriminative indicators.
6. Determining k – elbow curve and silhouette analysis identify the optimal number of clusters.
7. K‑means clustering – partition 82 exchanges and record cluster labels.
8. Random‑forest modelling – learn the relationship between the seven indicators and cluster membership.
9. SHAP analysis – derive global and local Shapley values to quantify how each indicator drives cluster assignment.
10. Reporting – visualise clusters, importance rankings and partial dependence.

## Results and discussion

The analysis of global stock exchanges was initiated through the application of clustering techniques to reveal inherent groupings within the dataset. The k-means algorithm was employed to identify these clusters, with the number of clusters (k) being a crucial parameter in defining the cohesion and separation of the resulting groups. The evaluation of clustering effectiveness was guided by two key diagnostic metrics: the silhouette coefficient and the distortion score. These metrics provided complementary perspectives on the quality of the clustering and were instrumental in determining the most appropriate number of clusters.

The initial phase of this study involved a broader set of variables intended to capture various dimensions of stock exchange performance and characteristics. These variables included measures such as market capitalization, trading volume, the number of listed companies, and other financial indicators. However, to enhance the interpretability and performance of the clustering, a variable selection process was conducted. This process aimed to maximize the silhouette coefficient, ensuring that only the most relevant variables contributing to meaningful cluster separation were retained. Through iterative testing and analysis, variables were carefully evaluated based on their impact on the clustering structure. Variables that did not significantly enhance the silhouette score were excluded from the final set, leading to a more cohesive and well-defined clustering outcome. This selection process ensured that the identified clusters were based on variables with the greatest explanatory power, improving the quality of the clustering and allowing for clearer insights into the underlying relationships within the data.

The figures below display five SHAP summary plots—one for each cluster produced by our gradient-boosting model. Every plot consists of two coordinated panels that must be read together. Left-hand panel (*beeswarm*). Each dot corresponds to a single stock exchange. Its horizontal position is the Shapley value (φ) for the feature on that row and therefore indicates whether the feature pushes the exchange towards the cluster (φ>0), pulls it away (φ<0), or has negligible influence (φ=0). The colour of the dot encodes the measured value of the feature: blue signifies a low value, red a high one. The vertical spread of dots shows how much the feature’s impact varies across exchanges—wide dispersion signals heterogeneity, while a compact band indicates a consistent effect.

Right-hand panel (bar chart of mean|φ|). Bars rank the same features by their mean absolute Shapley value; longer bars mark predictors that, on average, exert the greatest influence on cluster assignment, whereas shorter bars denote variables of limited importance.

A practical reading sequence is therefore:

1. Scan the right-hand bars to identify the dominant predictors for the cluster.
2. Consult the beeswarm to determine the direction (positive or negative), magnitude, and consistency of each predictor’s effect across individual exchanges.

All subsequent SHAP figures share this visual grammar. Once familiar with the framework, one can move through clusters 1–5 using the same two-step interpretation strategy described above.

**Fig 1:**
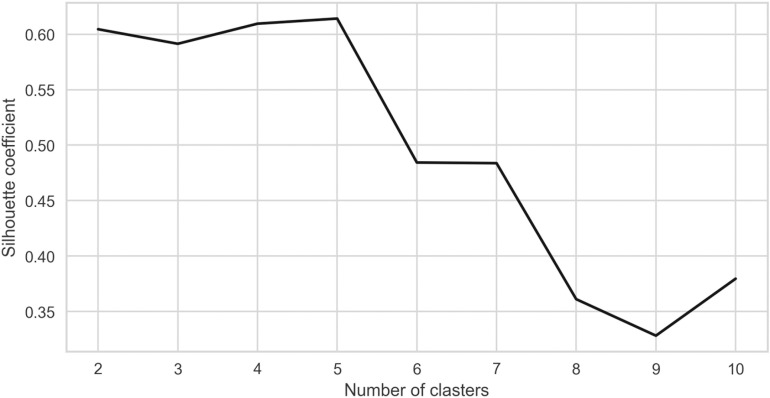
Silhouette coefficient vs. number of clusters.

Fig 1 offers a comprehensive overview of how the average silhouette coefficient varies with different values of k. The silhouette coefficient measures the degree of similarity between an observation and its assigned cluster compared to other clusters. A higher value indicates that the object is well-matched to its cluster and poorly matched to neighboring clusters, suggesting that the clustering structure is strong and distinct. As shown in the figure, the silhouette coefficient reaches its peak when k equals 5, with a value close to 0.7. This peak indicates that clustering the data into five groups results in the most cohesive and well-separated clusters. When the number of clusters is increased beyond five, the silhouette coefficient decreases significantly, indicating a reduction in cluster quality and an increased likelihood of overlap or poorly defined boundaries.

This insight is significant as it suggests that attempting to create more than five clusters results in diminishing returns in terms of clarity and separation, underscoring the robustness of k = 5 as the optimal choice. Clustering with fewer than five clusters results in a loss of granularity, failing to capture the complexity of the data and potentially grouping dissimilar exchanges together.

**Fig 2:**
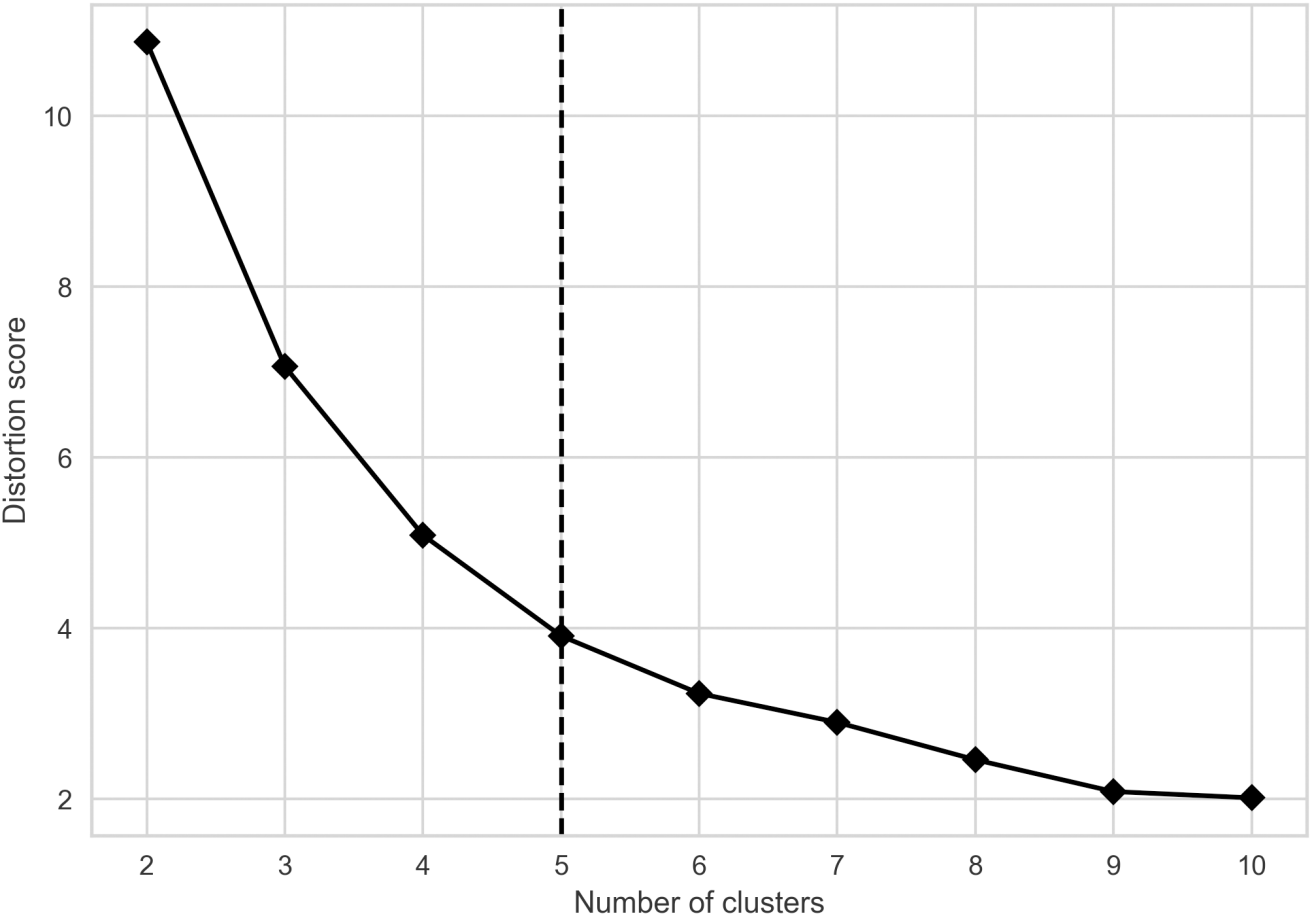
Distortion score vs. number of clusters.

Fig 2 complements the analysis presented in Fig 1 by illustrating the distortion score, which measures the compactness of the clusters. The distortion score is defined as the sum of squared distances from each observation to its cluster centroid. A lower score indicates that points within a cluster are closer to each other, signifying better cohesion. The plot in Fig 2 reveals a clear “elbow” at k = 5, marked by the dashed vertical line. This elbow indicates a point beyond which the addition of more clusters results in a significantly smaller decrease in the distortion score, suggesting that the incremental gain in cohesion does not justify the increased complexity of having additional clusters.

The convergence of results from both figures solidifies the decision to select k = 5 as the optimal number of clusters for this analysis. The silhouette coefficient in Fig 1 confirms that five clusters maintain a reasonable structure with distinct separation, while Fig 2 indicates that the reduction in distortion is meaningful up to k = 5 but becomes marginal thereafter.

These findings lay the groundwork for a deeper exploration of the characteristics of each cluster. Identifying five distinct groups of stock exchanges based on key financial and operational metrics enables further investigation into the unique attributes and strategic positions of these clusters in the global financial landscape. This comprehensive approach ensures that the analysis not only captures the overall distribution but also emphasizes the distinctiveness of each identified cluster. In subsequent sections, an analysis of the composition and features of these clusters will provide insights into the factors driving the development and competitiveness of stock exchanges worldwide.

Following the clustering analysis, which identified five distinct groups of stock exchanges, the focus shifted to interpreting the characteristics of each cluster through a classification model supported by SHAP (SHapley Additive exPlanations) values. This section presents an in-depth analysis of cluster 1, which comprises exchanges with notable market influence and significant trading activity, though they do not reach the scale of the world’s most dominant markets. Notable exchanges in cluster 1 include the Taipei Exchange, Korea Exchange, Borsa Istanbul, The Stock Exchange of Thailand, TMX Group, ASX Australian Securities Exchange, Japan Exchange Group, Taiwan Stock Exchange, Nasdaq Nordic and Baltics, National Stock Exchange of India, B3 - Brasil Bolsa Balcão, Euronext, and Deutsche Boerse AG. These exchanges serve as crucial financial hubs within their respective regions and play an integral role in global trading activity.

**Fig 3:**
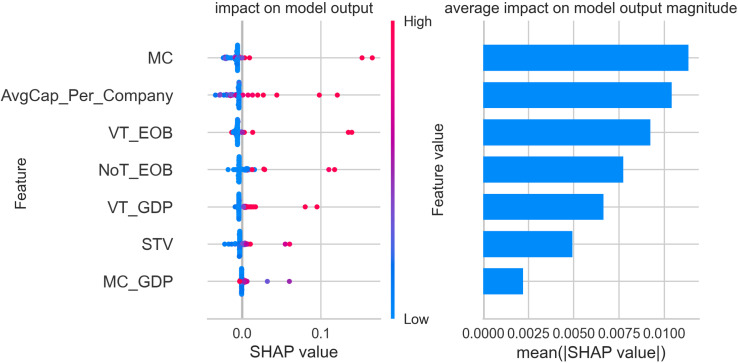
SHAP feature importance and impact plot – cluster 1.

Fig 3 presents the SHAP value analysis for cluster 1, offering insights into the significance of various financial features in classifying stock exchanges into this group. The left panel illustrates the distribution of SHAP values for each feature, showing their impact on the model’s predictions, while the right panel displays the average SHAP value magnitude, emphasizing the overall contribution of each feature to the classification process.

The analysis reveals that market capitalization (MC) is the most influential feature for determining whether an exchange belongs to cluster 1. High SHAP values associated with MC indicate that exchanges with substantial total market size are more likely to be classified within this group. This observation aligns with the inclusion of significant markets such as Euronext and Deutsche Boerse AG, which have robust market capitalizations and serve as key financial centers. Average capitalization per listed company (AvgCap_Per_Company) is another prominent feature in defining cluster 1. SHAP values show that exchanges with higher average company size are more likely to be associated with this cluster. This suggests that the cluster comprises markets where well-capitalized companies play a substantial role, even if the overall number of listed companies might not be as high as in the largest global markets. Trading activity, represented by value traded (VT_EOB) and the number of trades (NoT_EOB), also plays a significant role in the classification of cluster 1. High SHAP values for these features indicate that exchanges with high trading volumes and a substantial number of transactions are characteristic of this group. These findings underline the active and liquid nature of the markets in cluster 1, showcasing their importance in regional and international finance. The value traded to GDP ratio (VT_GDP), although less impactful than market capitalization or average capitalization per company, still contributes meaningfully to the classification. This feature indicates that exchanges in cluster 1 maintain a notable level of trading activity relative to the size of their national economies, reinforcing their role as significant financial platforms. Share turnover velocity (STV) and the market capitalization to GDP ratio (MC_GDP) appear with lower average SHAP values, yet they provide additional context in the classification. STV, which measures the trading frequency relative to market size, highlights the liquidity levels, while MC_GDP indicates the relative importance of the stock market within the broader economy.

The findings confirm that cluster 1 is characterized by exchanges with substantial market capitalization, a significant average company size, and active trading environments. These attributes position them as influential players in the global financial ecosystem, marked by their strong regional presence and economic impact, albeit not on par with the largest, most dominant global exchanges. This detailed SHAP analysis provides a clear view of the distinguishing features that define cluster 1 and its exchanges’ strategic roles within the global market structure.

**Fig 4:**
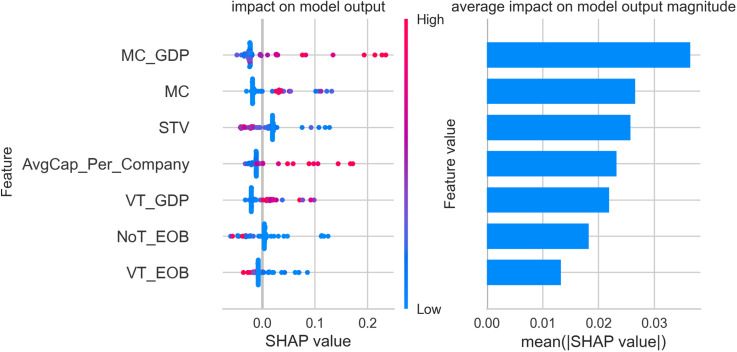
SHAP feature importance and impact plot – cluster 2.

Fig 4 provides an in-depth SHAP value analysis for cluster 2, illustrating the key financial features that define the classification of stock exchanges within this group. The market capitalization to GDP ratio (MC_GDP) emerges as the most significant feature for cluster 2, with high SHAP values indicating that exchanges with substantial market capitalization relative to their domestic GDP are more likely to be part of this cluster. This underscores the essential economic role these exchanges play within their respective countries, exemplified by the Tehran Stock Exchange, Johannesburg Stock Exchange, and Abu Dhabi Securities Exchange.

Market capitalization (MC) is another critical feature contributing to the classification of cluster 2. High SHAP values for this feature suggest that exchanges with significant total market sizes, such as the SIX Swiss Exchange and the Saudi Exchange (Tadawul), are central to this group. These exchanges are marked by their robust market presence, reflecting their importance as substantial financial centers. Share turnover velocity (STV) also stands out as a defining characteristic of cluster 2. SHAP analysis shows that high trading frequency relative to market size is a common feature among these exchanges, pointing to their active trading environments and liquidity. This highlights the dynamic market conditions that contribute to their classification. Average capitalization per listed company (AvgCap_Per_Company) contributes meaningfully to the cluster, indicating that markets with larger average company sizes are likely members of cluster 2. This is reflected in exchanges such as Abu Dhabi Securities Exchange and SIX Swiss Exchange, where well-capitalized firms dominate the market landscape. The value traded to GDP ratio (VT_GDP), though less impactful than the leading features, still plays a role in the classification. It suggests that exchanges in cluster 2 maintain trading activity that is significant relative to their economic scale, reinforcing their roles as active and vital components of their domestic financial systems. Trading volume metrics, including the number of trades (NoT_EOB) and value traded (VT_EOB), while showing moderate mean SHAP values, remain relevant to the classification of cluster 2. These features illustrate that exchanges within this cluster, such as the Saudi Exchange (Tadawul) and Johannesburg Stock Exchange, engage in substantial trading activity, setting them apart from smaller or less active markets.

Overall, the findings indicate that cluster 2 is characterized by exchanges with significant market capitalization, strong economic relevance within their national economies, and active trading environments. Notable members of this cluster include the Tehran Stock Exchange, Johannesburg Stock Exchange, Abu Dhabi Securities Exchange, SIX Swiss Exchange, and Saudi Exchange (Tadawul). Additionally, exchanges such as the Tel-Aviv Stock Exchange, Kuwait Stock Exchange, and Qatar Stock Exchange may share similar characteristics, underscoring the regional prominence and strategic importance of the exchanges within cluster 2 in the global financial ecosystem.

**Fig 5:**
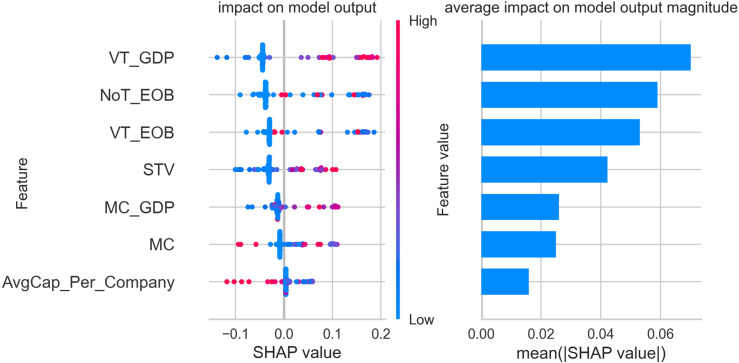
SHAP feature importance and impact plot – cluster 3.

Fig 5 provides an in-depth SHAP value analysis for cluster 3, identifying the key financial features that drive the classification of stock exchanges in this group. The most significant feature for cluster 3 is the value traded to GDP ratio (VT_GDP). High SHAP values for this feature indicate that exchanges with a considerable volume of trading relative to the size of their domestic economy are more likely to be classified in this cluster. This characteristic underscores the substantial trading activity that defines exchanges such as Nasdaq (US) and NYSE.

The number of trades (NoT_EOB) also plays a crucial role in defining cluster 3. High SHAP values associated with this feature highlight that exchanges with a high frequency of transactions are characteristic of this group. This reinforces the active trading environment and high liquidity that are hallmarks of cluster 3, emphasizing the dynamic market operations of its members. Value traded (VT_EOB) is another important feature, with SHAP values showing that exchanges with significant trading volumes are prominent in this cluster. This further illustrates the scale of financial activity present in cluster 3, which is consistent with the profiles of major global exchanges like Nasdaq and NYSE, known for their substantial trading activities and market leadership. Share turnover velocity (STV) also contributes notably to the classification, indicating that the frequency at which shares change hands is an important aspect of these exchanges. This feature underscores the efficiency and liquidity of the markets within cluster 3, where trading activity is robust and continuous. Market capitalization to GDP ratio (MC_GDP) and market capitalization (MC) show moderate SHAP values in the analysis. This suggests that while these features contribute to the identification of cluster 3, they are not as defining as the trading activity metrics. Nonetheless, the substantial size of these exchanges in terms of market capitalization supports their classification within this cluster, marking them as dominant financial powerhouses. Average capitalization per listed company (AvgCap_Per_Company) has a lower mean SHAP value but still provides context. Its inclusion indicates that while the average company size is significant, it is not the primary factor that differentiates this cluster.

Overall, the findings for cluster 3 show that it is defined by exchanges with extremely high trading activity, frequent transactions, and substantial market engagement relative to their domestic economies. Key members of this cluster include Nasdaq (US) and NYSE, both exemplifying these characteristics through their leadership in trading volumes, liquidity, and economic influence. This analysis highlights the position of cluster 3 as comprising the most dynamic and influential stock exchanges within the global financial ecosystem.

**Fig 6:**
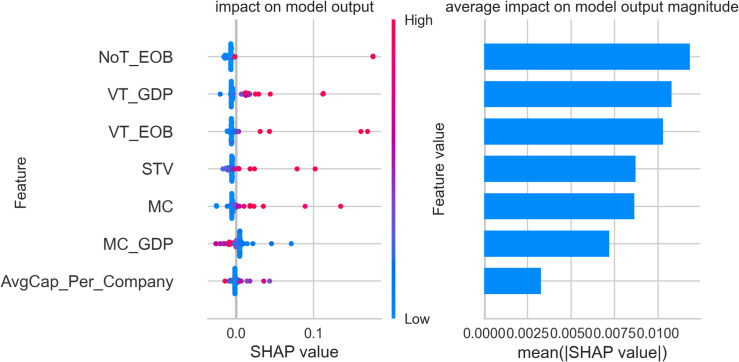
SHAP feature importance and impact plot – cluster 4.

Fig 6 presents the SHAP value analysis for cluster 4, revealing the key features that contribute to the classification of this diverse group of stock exchanges. This cluster encompasses a broad range of markets, representing a significant portion of the global financial landscape. Notable exchanges within this cluster include the Warsaw Stock Exchange, LSE Group London Stock Exchange, Moscow Exchange, and the Bolsa Mexicana de Valores, along with smaller regional markets such as the Armenia Securities Exchange and the Namibian Stock Exchange.

The analysis shows that the number of trades (NoT_EOB) is the most significant feature influencing the classification of exchanges into cluster 4. High SHAP values for this feature indicate that trading frequency is a key characteristic of these markets. This suggests that while the exchanges in this cluster vary in size and influence, they are generally defined by substantial trading activity relative to other features. The value traded to GDP ratio (VT_GDP) follows closely in importance, highlighting that many exchanges in this group maintain a meaningful level of trading activity compared to the size of their domestic economies. This feature reflects the activity levels and economic engagement of these markets, ranging from larger exchanges like the LSE Group London Stock Exchange to smaller ones like the Ghana Stock Exchange. Value traded (VT_EOB) and share turnover velocity (STV) are also significant contributors to the classification. SHAP values for these features emphasize that exchanges in cluster 4 exhibit varied degrees of liquidity and market dynamism. This is consistent with the diversity within the cluster, which includes established exchanges with high trading volumes and others that, while active, have lower overall liquidity. Market capitalization (MC) and market capitalization to GDP ratio (MC_GDP) show moderate SHAP values, suggesting that while these metrics are relevant, they are not the primary differentiators for cluster 4. This aligns with the composition of the cluster, which includes exchanges with varying levels of market size—from major regional players like the Warsaw Stock Exchange to smaller markets like the Cyprus Stock Exchange. Average capitalization per listed company (AvgCap_Per_Company) has the lowest mean SHAP value among the key features, indicating that while the average company size plays a role, it is not a significant factor in distinguishing this cluster. This reflects the heterogeneous nature of the exchanges in cluster 4, which range from markets with prominent large-cap companies to those with a mix of smaller entities.

Overall, cluster 4 encompasses a wide spectrum of stock exchanges characterized by active trading environments and significant economic engagement, yet varied in terms of market size and liquidity. Exchanges such as the LSE Group London Stock Exchange and Moscow Exchange are balanced by numerous smaller or regionally focused markets, making this cluster the broadest in terms of diversity and representation within the global financial landscape. This analysis underscores the role of cluster 4 as encompassing the majority of exchanges that, while influential and active, do not fall into the specialized categories represented by other clusters.

**Fig 7:**
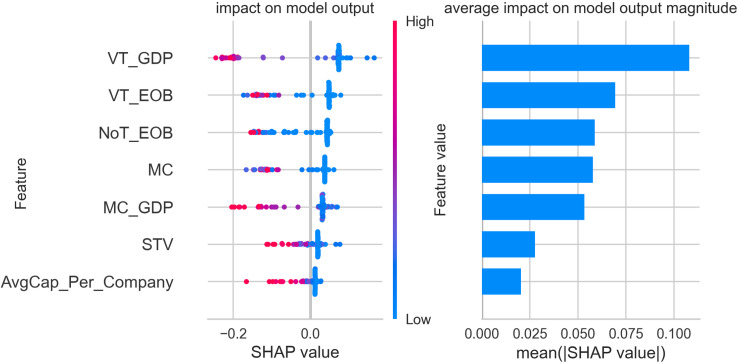
SHAP feature importance and impact plot – cluster 5.

Fig 7 presents the SHAP value analysis for cluster 5, highlighting the defining features of this unique group of stock exchanges. Cluster 5 is composed of major Chinese exchanges, notably the Shenzhen Stock Exchange and the Shanghai Stock Exchange, which are characterized by their considerable scale and economic influence within China and globally.

The most significant feature for cluster 5 is the value traded to GDP ratio (VT_GDP). High SHAP values associated with this feature indicate that these exchanges have a high trading volume relative to their domestic economic output, underscoring their critical role in facilitating substantial financial activity within a rapidly growing economy. Value traded (VT_EOB) follows closely in importance, emphasizing that the total trading volume is a key characteristic of the exchanges in cluster 5. This aligns with the known high levels of activity on the Shenzhen and Shanghai exchanges, which consistently report large trading volumes due to the high participation of domestic investors and their impact on the global financial landscape. The number of trades (NoT_EOB) also contributes significantly to the classification, reflecting the high frequency of transactions on these exchanges. This feature highlights the dynamic nature of trading in cluster 5, characterized by active engagement and liquidity that distinguishes these markets from others. Market capitalization (MC) and the market capitalization to GDP ratio (MC_GDP) show moderate SHAP values, indicating that while these features are influential, they are not the primary factors driving the classification. However, they do point to the sheer size and economic weight of the Shanghai and Shenzhen Stock Exchanges, both of which rank among the largest globally by market capitalization. Share turnover velocity (STV) has a slightly lower impact but still adds context to the cluster’s classification. This feature reflects the rapid pace at which shares change hands on these exchanges, underscoring the liquidity and trading efficiency that are hallmarks of these markets. Average capitalization per listed company (AvgCap_Per_Company) shows the lowest mean SHAP value in this analysis, indicating that while company size is relevant, it is not a key differentiator for cluster 5. This aligns with the diverse range of companies, from large state-owned enterprises to smaller, high-growth firms, listed on these exchanges.

Overall, cluster 5 is defined by its high trading volume, significant frequency of transactions, and robust economic involvement as reflected in the value traded to GDP ratio. The Shenzhen Stock Exchange and Shanghai Stock Exchange exemplify these characteristics, positioning themselves as pivotal financial hubs within China and critical players in the global market. The findings underline the strategic importance of these exchanges in both regional and international financial ecosystems, marked by their unique scale and dynamic trading activity.

**Fig 8:**
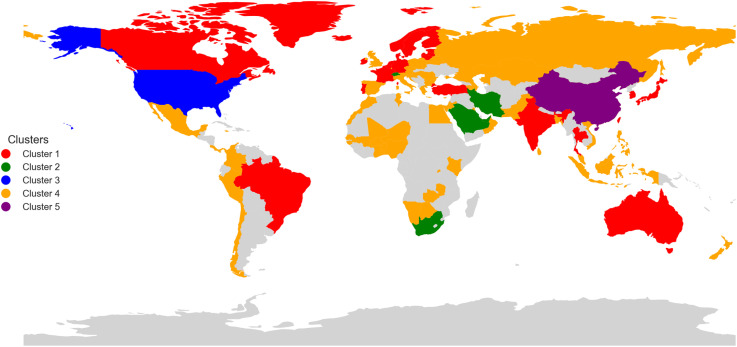
Geographic distribution of analysed stock exchanges. The map was created by the authors using Python’s Geopandas library and public domain data from Natural Earth (http://www.naturalearthdata.com). No copyright restrictions apply.

Fig 8 provides a comprehensive visual representation of the global distribution of stock exchanges, grouped into five distinct clusters. This classification highlights the varied levels of economic influence and trading activity across different regions. North America is prominently featured with significant financial centers, showcasing its position as a key player in the global economy. Europe displays a mix of dominant exchanges and diverse smaller markets, indicating its complex and interconnected financial landscape. The presence of clusters across Africa, Asia, and Oceania emphasizes the importance of regional hubs that facilitate economic activity and connect global trade. Notably, East Asia stands out with its unique concentration of significant exchanges, reflecting the region’s growing economic power and dynamic market activity. Fig 8 underscores the varied nature of stock exchanges worldwide, from globally leading markets to smaller but active financial centers that play vital roles in their respective regions.

To provide a clear overview of the clustering results, Table 1 summarizes the attributes and notable members of each cluster, helping to contextualize their roles within the global financial landscape. The table highlights the diverse nature of these clusters, illustrating the varying levels of market influence and trading activity—from globally dominant exchanges with the highest market presence to smaller, regionally significant markets that play vital roles within their local economies.

**Table 1:** Summary of cluster characteristics and notable exchanges.

Cluster	Key characteristics	Notable exchanges
Cluster 1	Significant market influence, strong market capitalization, active trading environments; not at the scale of the largest global exchanges	Euronext, Deutsche Boerse AG, ASX Australian Securities Exchange, TMX Group, Japan Exchange Group, Taiwan Stock Exchange
Cluster 2	High economic relevance within their domestic markets, strong trading activity relative to GDP, significant market size	Saudi Exchange (Tadawul), Johannesburg Stock Exchange, SIX Swiss Exchange, Abu Dhabi Securities Exchange, Tehran Stock Exchange
Cluster 3	Globally dominant with the highest trading volumes, leading market capitalization, highly active and liquid markets	NYSE, Nasdaq (US)
Cluster 4	Diverse group with varying levels of trading activity, market sizes, and economic engagement; includes regional leaders and smaller, active exchanges	Warsaw Stock Exchange, LSE Group London Stock Exchange, Moscow Exchange, Bolsa Mexicana de Valores, Athens Stock Exchange, Philippine Stock Exchange, Vienna Stock Exchange, BME Spanish Exchanges
Cluster 5	High trading volume, significant regional influence in East Asia, active trading environments, marked by both large-cap and smaller growth companies	Shanghai Stock Exchange, Shenzhen Stock Exchange

This table underscores the range of characteristics present across the clusters, from the highest levels of market activity and global reach to more specialized and regionally influential exchanges. It effectively illustrates the unique roles these clusters play in the global financial ecosystem, reinforcing the varied contributions of exchanges within their respective markets.

A set of well-established frameworks in international finance and political economy help explain why the exchanges in Table 1 map onto the five empirical clusters displayed in Fig 8. First, the gravity model of cross-border portfolio flows predicts that trading volumes rise with the joint economic “mass” of two markets and fall with informational and institutional frictions between them [77]. Cluster 3 (NYSE and Nasdaq) sits at the model’s large-mass/low-friction extreme: the United States combines outsized GDP with deep, highly liberalised capital markets. In contrast, Cluster 5 is concentrated in mainland China, where large economic mass coexists with capital-account controls and a state-centric governance structure [78]. This polarity is consistent with the open-economy trilemma: countries cannot simultaneously enjoy monetary autonomy, exchange-rate stability, and full financial openness, so they sort into groups according to the policy mix they privilege [79].

Institutional similarity and legal origin provide a second clustering mechanism. Exchanges in Cluster 1 (e.g., Euronext, Deutsche Boerse, TMX, ASX, JPX) operate in common-law or Germanic civil-law jurisdictions with strong shareholder protections and rigorous disclosure rules, which foster comparable liquidity levels [25]. By contrast, Cluster 2 comprises resource-rich monarchies or late-industrialising mixed systems (Saudi Arabia, UAE, South Africa, Iran, Switzerland). Here, state–capital dynamics dominate: free float is limited, cross-listing is rare, and market openness remains intermediate [78].

A third layer is geo-economic embeddedness. Cluster 4 is heterogeneous, yet its members share peripheral—but institutionally tethered—positions in larger regional blocs: EU enlargement (Warsaw, Vienna, Athens), USMCA spill-overs (Mexico), or Eurasian Economic Union linkages (Moscow). In world-systems terminology they function as semi-peripheral nodes that intermediate capital between core and periphery [80].

Adding further nuance, recent work on financial-network topology shows that markets sharing common trading technology, settlement infrastructure, or ownership stakes form densely connected “communities” that transmit volatility more intensely within than across groups [81]. These meso-level ties explain why geographically distant venues—such as Australia and Germany—display stronger similarities to one another than to some nearer neighbours.

Path-dependence and cultural proximity also matter. Historical ties (colonial links, common legal codes, and language) reduce information asymmetries and foster bilateral listings, thereby increasing the probability that exchanges fall into the same cluster [82,83]. This mechanism helps rationalise the placement of London and Johannesburg—former metropole and colony—within neighbouring clusters despite their different continents.

Finally, the clusters echo broader trajectories of financial globalisation and de-globalisation. Long-run data show that integration surges in waves punctuated by crises and regulatory resets [84]. Cluster membership therefore captures not only static fundamentals but also shared exposure to global integration cycles: the strongly outward-oriented exchanges in Clusters 1 and 3 gained disproportionately during the 1990s liberalisation wave, whereas the more insulated members of Clusters 2 and 5 preserved tighter policy buffers after the 2008 crisis. These dynamics underline that the pattern in Fig 8 represents enduring combinations of market scale, institutional quality, policy choice and geo-economic alignment rather than a purely data-driven artefact.

## Conclusion

This study undertook a comprehensive analysis of global stock exchanges to identify development patterns and understand the factors influencing their positions within the international financial system. By integrating k-means clustering, random forest classification, and SHAP value interpretation, we achieved a nuanced understanding of the similarities and differences among 82 stock exchanges worldwide. The primary aim was to uncover inherent groupings based on key financial and operational metrics and to interpret the underlying factors contributing to these clusters.

The clustering analysis successfully identified five distinct groups of stock exchanges, each characterized by specific features related to market capitalization, trading activity, liquidity, and economic influence. The optimal number of clusters was determined to be five, based on the silhouette coefficient and distortion score metrics, ensuring that the clusters were cohesive and well-separated. This optimal clustering allowed for a meaningful interpretation of the underlying structures within the data without unnecessary complexity.

Relating back to the aim of the study, the identification of these clusters provided valuable insights into how different financial indicators influence the positioning of stock exchanges in the global market. The findings highlight the diverse nature of stock exchanges, ranging from globally dominant markets with high trading volumes and market capitalization to smaller, regionally significant exchanges that play vital roles within their local economies.

The SHAP value analysis revealed that features such as market capitalization, trading activity metrics, and economic relevance were the most influential factors in determining cluster membership. Market capitalization and average capitalization per listed company emerged as critical in distinguishing exchanges with significant market influence. Trading activity indicators, including the value traded to GDP ratio and the number of trades, were pivotal in identifying exchanges characterized by dynamic market operations and high liquidity. The market capitalization to GDP ratio underscored the essential role some exchanges play within their domestic economies, reflecting their importance as substantial financial centers.

One of the notable conclusions drawn from this study is the potential benefits of exchange consolidation in enhancing competitiveness and efficiency. The findings suggest that stock exchanges formed through the consolidation of smaller exchanges can create more substantial and competitive markets with increased liquidity, reduced fragmentation, and enhanced access for investors and issuers. Examples of such exchanges include Euronext and Nasdaq Nordic and Baltic. Euronext serves as a prime example of successful consolidation, bringing together markets from several European countries, including France, the Netherlands, Belgium, Portugal, and Ireland. This integration has led to increased liquidity, improved market efficiency, and a broader range of financial products and services available to market participants. Similarly, Nasdaq Nordic and Baltic exchanges have benefited from uniting exchanges from Sweden, Denmark, Finland, Iceland, and the Baltic states, establishing a more robust market presence in Northern Europe. This consolidation has facilitated cross-border trading, attracted more international investment, and enhanced technological capabilities.

The consolidation of exchanges can lead to increased liquidity, economies of scale, and enhanced competitiveness on a global scale. By combining resources and harmonizing regulatory frameworks, consolidated exchanges can offer more efficient services, reduce transaction costs, and provide a wider array of investment opportunities. This approach can be particularly beneficial for smaller or emerging markets seeking to enhance their global reach and attractiveness to international investors.

The implications of these findings are significant for policymakers, regulators, and market participants. Understanding the factors that influence the development and competitiveness of stock exchanges can inform strategies aimed at strengthening financial infrastructure and promoting sustainable economic growth. The study underscores the importance of key financial indicators in shaping the global financial landscape and highlights the potential of strategic consolidation as a means to enhance market efficiency.

Furthermore, the methodological framework employed in this study demonstrates the effectiveness of integrating clustering algorithms with machine learning classification and interpretability techniques. The use of k-means clustering provided a data-driven approach to uncover natural groupings within the exchanges. The random forest classifier facilitated the modeling of relationships between features and cluster labels, enabling the computation of SHAP values for interpretability. This integrative approach allowed for a comprehensive analysis that not only identified distinct clusters but also provided insights into the underlying factors influencing stock exchange development and competitiveness.

While the study provides valuable insights, it is essential to acknowledge certain limitations. The analysis was based on data from 2023, and changes in market conditions or data reporting practices could affect the results. Additionally, although the variable selection aimed to maximize the silhouette score, other relevant variables may further enhance the clustering and interpretation. Including more exchanges or focusing on specific regions could yield additional insights and refine the conclusions drawn.

Future research could proceed along several complementary avenues. First, the attribution analysis itself can be deepened by experimenting with alternative explainers—most notably the Shapley-Lorenz value, which rescales the classical Shapley scores so that they sum to one and thus enables scale-free comparisons across clusters and models [85]. Other methods (e.g., Integrated Gradients or permutation importance) could serve as robustness checks for the SHAP‐based findings. Second, our static clustering could be extended with dynamic or regime-switching models to track how groups of exchanges evolve over time. Third, the study’s thematic focus may shift from classification to consolidation: analysing the long-term effects of exchange mergers on market efficiency, investor behaviour and broader economic development; assessing the associated regulatory and antitrust challenges, including the harmonisation of listing and disclosure standards; and evaluating how consolidated entities can exploit emerging technologies—advanced matching engines, real-time surveillance, cybersecurity tools and data-analytics platforms—to bolster competitiveness and systemic resilience. Collectively, these directions would not only refine the explanatory toolkit but also contextualise cluster membership within the strategic, regulatory and technological forces shaping global exchange ecosystems.

In conclusion, this study successfully identified development patterns among global stock exchanges and highlighted the factors influencing their competitiveness and positioning within the global financial system. The integration of clustering, classification, and interpretability techniques provided a comprehensive understanding of the exchanges’ characteristics. The findings emphasize the strategic importance of exchange consolidation as a means to enhance market efficiency, liquidity, and global competitiveness. By shedding light on the dynamics of stock exchange development, this study contributes to the broader discourse on financial market integration and offers actionable insights for stakeholders aiming to strengthen financial infrastructure and promote economic prosperity.
